# Sea Surface Height Estimation with Multi-GNSS and Wavelet De-noising

**DOI:** 10.1038/s41598-019-51802-9

**Published:** 2019-10-23

**Authors:** Fade Chen, Lilong Liu, Fei Guo

**Affiliations:** 1 School of Geodesy and Geomatics, Wuhan University, Wuhan, 430079 China; 2 College of Geomatic Engineering and Geoinformatics, Guilin University of Technology, Guilin, 541004 China; 3 Guangxi Key Laboratory of Spatial Information and Geomatics, Guilin, 541004 China

**Keywords:** Environmental sciences, Physical oceanography

## Abstract

This paper presents a new sea surface height (SSH) estimation using GNSS reflectometry (GNSS-R). It is a cost-effective remote sensing technique and owns long-term stability besides high temporal and spatial resolution. Initial *in-situ* SSH estimates are first produced by using the SNR data of BDS (L1, L5, L7), GPS (L1, L2, L5), and GLONASS (L1, L2), of MAYG station, which is located in Mayotte, France near the Indian Ocean. The results of observation data over a period of seven days showed that the root mean square error (RMSE) of SSH estimation is about 32 cm and the correlation coefficient is about 0.83. The tidal waveform is reconstructed based on the initial SSH estimates by utilizing the wavelet de-noising technique. By comparing the tide gauge measurements with the reconstructed tidal waveform at SSH estimation instants, the SSH estimation errors can be obtained. The results demonstrate that the correlation coefficient and RMSE of the wavelet de-noising based SSH estimation is 0.95 and 19 cm, respectively. Compared with the initial estimation results, the correlation coefficient is improved by about 14.5%, while the RMSE is reduced by 40.6%.

## Introduction

Obtaining accurate Sea Surface Height (SSH) is significant for human living, especially for those live along ocean coasts. Furthermore, it is also important to coastal ecosystems and coastal morphology^1,2^. SSH has been obtained primarily with tide gauges during the last centuries. While for the last three decades, satellite altimetry has been the dominating technique^3–5^. The satellite altimetry has unique advantages in monitoring sea surface topography in open ocean research such as ocean circulation^6^. However, due to tidal aliasing effect, it’s spatial and temporal resolution are insufficient to observe complex and rapidly changing dynamics which makes it difficult to use near the coast^7^. Meanwhile, tide gauges are affected by both sea level and land surface changes since the measurements are related to a benchmark on the land, where they were established. For these reasons, it is difficult to use traditional tide gauges for sea-level studies in tectonically active areas or applications related to changes in the global ocean volume, e.g., the global sea level budget^8^. These applications need absolute observations of sea level, in other words, measurements with respect to a terrestrial reference frame^9^.

In recent years, the reflected signals of Global Navigation Satellite System (GNSS) are used to retrieve a range of geophysical parameters such as SSH, soil moisture, ocean wind speed, etc.^10–18^. This technique is also termed GNSS-Reflectometry (GNSS-R), which exploits conventional receivers or low-cost, low-mass, and low-power characteristics of GNSS-R receivers. Different GNSS-R based methods have been proposed in the literature to measure a range of geophysical parameters. Basically, either a single antenna or two separate antennas are used for the direct and reflected signal reception. A number of researchers have investigated GNSS-based ocean surface altimetry by using the measurement of signal arrival time obtained by either mounting the receiver on an aircraft or fixing it on the ground such as on a bridge^19,20^. Anderson *et al*. first proposed to use the interference pattern in the recorded Signal-to-Noise Ratio (SNR) to estimate SSH^21^. Later, Larson *et al*. applied the SNR method to SSH of the nearby ocean during three months^22^. Santamaría-Gómez *et al*. developed an approach to extract SNR data dominated by sea-surface reflections and to remove SNR frequency changes caused by the dynamic sea surface^23^. They successfully demonstrated its ability to estimate local SSH and improved accuracy. Nevertheless, many of their studies used single frequency or single system. With constantly developing and perfecting of other navigation satellite systems such as Galileo and BDS, can we improve the accuracy of SSH estimation using multi frequencies or multi-systems? There are still many challenging problems to be resolved^24^.

In this paper, we propose an improvement of the method based on the SNR analysis of a single antenna. And the focus is on GNSS-R ocean surface altimetry based on the SNR data of multiple satellite constellations and multiple frequencies, namely BDS (L1, L5, L7), GPS (L1, L2, L5) and GLONASS (L1, L2). The purpose is to exploit measurements diversity to achieve a performance gain. The structure of the next part of this paper is organized as follows. Firstly, the fundamental theory of SSH GNSS-R and contains a wavelet analysis in detail are given. Thereafter the data introduction is presented. Then SSH estimation results by using individual GNSS-R and multi-GNSS-R are firstly shown. The SNR data collected by an IGS station are used for evaluation and analysis. Tide gauge observations are used as ground-truth data for performance comparison. Furthermore, the initial discrete SSH estimation results are used to reconstruct the tidal waveform by using wavelet de-noising technique. Finally, the concluding remarks are declared.

## Theory and Methods

### Methods of GNSS-R

**Figure 1:**
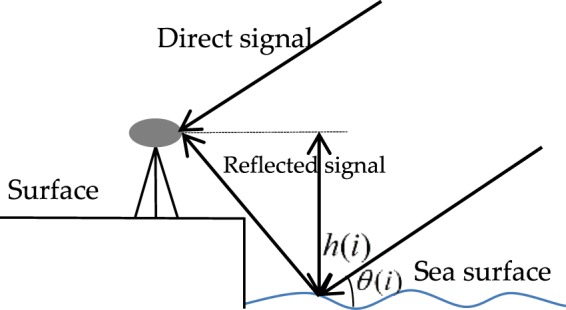
Diagram of GNSS-R for SSH variation.

Multipath propagation is one of the main GNSS error sources, which constrains high-precision positioning. The multipath caused by the difference of the phase between the direct and reflected signals will affect GNSS observations and give rise to oscillations in the observations. As a result, the SNR data recorded by a GNSS receiver with a single antenna will contain information about the interference, antenna height and hence SSH variation. Figure 1 shows the diagram of SSH estimation based on the SNR method. Here *h*(*i*) is the antenna height relative to reflection surface, and *θ*(*i*) is the angle between the direct signal and the instantaneous sea surface of a specific scattering point. In Fig. 1, the reflected signal has one excess phase delay when compared to the direct signal, which is related to the antenna height *h*(*i*).

**Figure 2:**
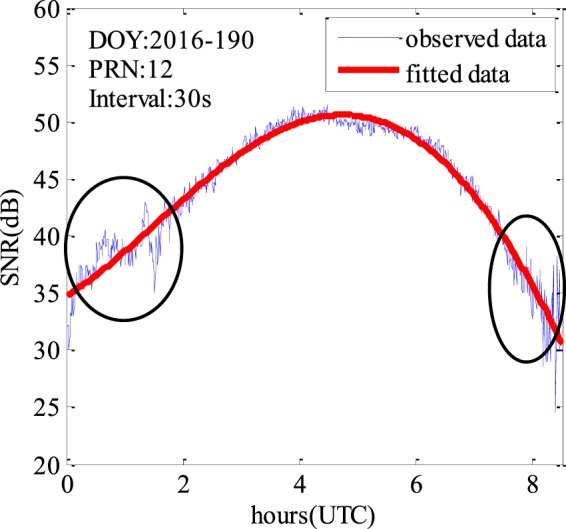
SNR variations observed by a BDS receiver.

Figure 2 shows an example of the SNR variations recorded by a BDS receiver. The thin blue fast-varying curve is the observed data, while the red thick smooth curve is the fitted SNR of the direct signal. As we can see, the multipath SNR is proportional to the difference between the observed curve and the fitted curve. It can be seen that the multipath SNR during the rising and setting of the satellite is significantly higher than that elsewhere, as shown in the two black ellipses.

SNR is one of the main observables of the GNSS receiver, which is mainly related to the satellite signal transmission power, antenna gain, and multipath effect. As the elevation angle increases, the antenna gain raises, subsequently, the direct signal SNR increases. In this paper, we use the observed SNR data from the GNSS receiver to estimate SSH. As described in^22^, the SNR is given by

1 $$\begin{array}{rcl}SNR & = & \frac{{A}^{2}}{2{P}_{n}}\\  & = & \frac{1}{2{P}_{n}}({A}_{d}^{2}+{A}_{r}^{2}+2{A}_{d}{A}_{r}\,\cos \,\delta \varphi (t))\end{array}$$

where *P*_*n*_ is the noise power, *A*_*d*_ indicates amplitude of direct signal, *A*_*r*_ refers amplitude of the reflected signal, *δϕ*(*t*) is the reflection excess phase with respect to direct phase (also known as the interferometric phase), given by

2 $$\delta \varphi (t)=\frac{4\pi h}{\lambda }\,\sin \,\theta (t)$$

where *h* stands for antenna height from the specular scattering point on the reflected surface, *λ* is the carrier wavelength, *θ*(*t*) represents the elevation angle. Since (i) GNSS antennas are designed to filter reflected signals, (ii) the reflected signal is attenuated upon reflection, and (iii) the azimuth angles we choose to make reflected signals come from the sea surface, we can assume that

3 $${A}_{d}\gg {A}_{r}$$

Therefore, the direct signal determines the overall trend of the SNR observations (see Fig. 2). So, we can use a low-order polynomial to fit the observed SNR data and then remove the trend. As a result, a detrended SNR time series is produced, which can be used to estimate the surface parameters such as SSH. As described in^22^, after removing the SNR trend, the remaining SNR can be approximated as

4 $$dSNR=A\,\cos (\frac{4\pi h}{\lambda }\,\sin \,\theta (t)+\phi )$$

where *φ* indicates a phase offset, *A* denotes the amplitude which is given by

5 $$A=\frac{{A}_{d}\cdot {A}_{r}}{{P}_{n}}$$

By defining *ω* = sin *θ* and *f* = 2*h*/*λ*, Eq. (4) can be rewritten as

6 $$dSNR=A\,\cos (2\pi f\omega +\phi )$$

Due to the tidal effect, the antenna height relative to the surface may change smoothly by around 0.72 m during half an hour at the selected observation station. Thus, the frequency of the detrended SNR signal varies gently with time. However, the mean frequency of the signal would be dominant, which basically corresponds to the antenna height at the middle of the observation time period.

From Eq. (6) and the definition of *f*, the antenna height is associated with the frequency of the detrended SNR signal by

7 $$h=f\lambda /2$$

Spectral analysis is applied to obtain the spectral peak frequency of the time series. In this paper, the Lomb-Scargle periodogram (LSP) method is used since it can handle unevenly spaced samples^22^. The recorded SNR data are typically evenly sampled in time, but the sine of elevation angle is not evenly distributed. The LSP is a well-known algorithm for detecting and characterizing periodicity in unevenly-sampled time-series, even more, has seen particularly wide use within the astronomy community^25^. As described in^25^, the LSP method calculates the power spectral density by

8 $${P}_{x}(f)=\frac{1}{2{\delta }^{2}}\{\frac{{[\sum ({X}_{i}-\overline{X})\cos \omega ({t}_{i}-\tau )]}^{2}}{\sum {\cos }^{2}\omega ({t}_{i}-\tau )}+\frac{{[\sum ({X}_{i}-\overline{X})\cos \omega ({t}_{i}-\tau )]}^{2}}{\sum {\sin }^{2}\omega ({t}_{i}-\tau )}\}$$

where $\overline{X}$ and *δ*^2^ are the mean and the variance of the observed sequence respectively, *ω* denotes the angular frequency, and *τ* symbolizes the phase which can be inferred by

9 $$\tan (2\omega \tau )=\sum \sin (2\omega {t}_{i})/\sum \cos (2\omega {t}_{i})$$

**Figure 3:**
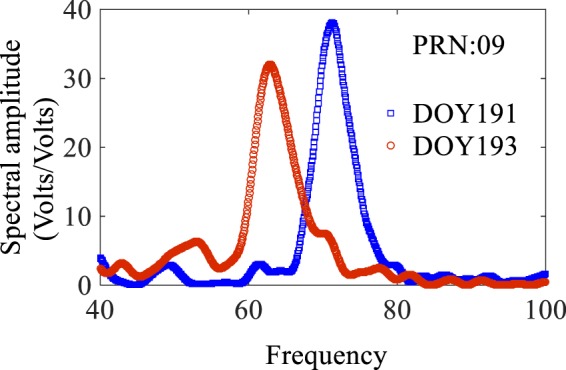
Detrended SNR spectrum produced by Lomb-Scargle periodogram analysis.

As an example of obtaining the peak frequency using LSP method, Fig. 3 shows the results of LSP analysis of the detrended SNR time series from the SNR data observed by BDS satellite PRN09 on two different days. The elevation angles change from 5° to 20° and from 20° to 5° for day of year (DOY) 191 and DOY 193, respectively. The peak spectral frequency can be clearly observed. The peak frequency difference is caused by the variation in the SSH over the two various observation periods. The estimated peak frequency, besides, Eq. (7) are applied to obtain the antenna height.

### Wavelet De-noising

The concept of wavelet was first proposed by Morlet in 1984^26^. Later, under the help of Grossmann, Morlet formalized the continuous wavelet transform (CWT)^27^ as follow

10 $${W}_{\psi }\,f(a,b)=\langle \,f,{\psi }_{a,b}\rangle ={a}^{-1/2}{\int }_{-\infty }^{+\infty }f(t)\overline{\psi }(\frac{t-b}{a})dt$$

where *a* indicates the scale parameter, *b* refers to the time parameter, *ψ*(*t*) is an analyzing wavelet, and $\overline{\psi }(\bullet )$ symbolizes the complex conjugate of *ψ*(•). The inverse transform of CWT is given by

11 $$f(t)=\frac{1}{{C}_{\psi }}{\int }_{-\infty }^{+\infty }\{{W}_{\psi }\,f(a,b)[{a}^{-1/2}\psi (\frac{t-b}{a})db]\}\frac{da}{{a}^{2}}$$

where *a*, *b* and *t* are continuous. However, they must be discretized in analyzing real data. The discrete method is called discrete wavelet transform (DWT). In practice, the scale *a* and the time *b* are discretized as following

12 $$a={a}_{0}^{j},\,b=k{a}_{0}^{j}{b}_{0}$$

where *j* and *k* are integers. And the continuous wavelet function *W*_*ψ*_*f*(*a*, *b*) become the DWT, given by

13 $$\begin{array}{ccc}{C}_{j,k}={\int }_{-\infty }^{+\infty }f(t){\overline{\psi }}_{j,k}(t)dt & with & {\psi }_{j,k}(t)={2}^{-j/2}({2}^{-j}t-k)\end{array}$$

The inverse transform of DWT is computed by

14 $$f(t)=\sum _{j\in Z}\sum _{K\in Z}{C}_{j,k}{\psi }_{j,k}(t)$$

**Figure 4:**
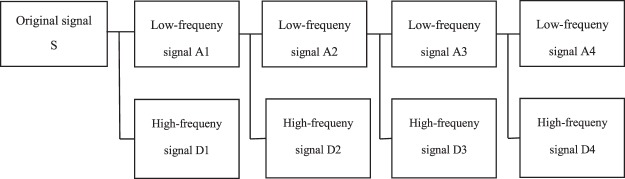
Wavelet decomposition filter model.

Mallat proposed a fast DWT algorithm in 1989^28^, which decompose and reconstruct the signal using the wavelet filters. The decomposition algorithm is given by

15 $$\begin{array}{rcl}{A}_{j}[\,f(t)] & = & \mathop{\sum _{k}H(2t-k){A}_{j-1}[\,f(t)]}\limits^{{A}_{0}[f(t)]=f(t)}\\ {D}_{j}[\,f(t)] & = & \sum _{k}G(2t-k){A}_{j-1}[\,f(t)]\end{array}\}$$

where *t* = 1, 2 …, *N* is the discrete time serial number, *N* denotes the signal length, *f*(*t*) symbolizes the original signal, *j* = 1, 2 …, *J* is the decomposition level, *J* is the maximum decomposition level; *H* and *G* are the wavelet low-frequency and high-frequency pass decomposition filters, respectively; *A*_*j*_ and *D*_*j*_ are the wavelet coefficients of *f*(*t*) in the low-frequency and the high-frequency part of the *J*th layer, respectively. Figure 4 shows the wavelet decomposition filter model.

**Figure 5:**
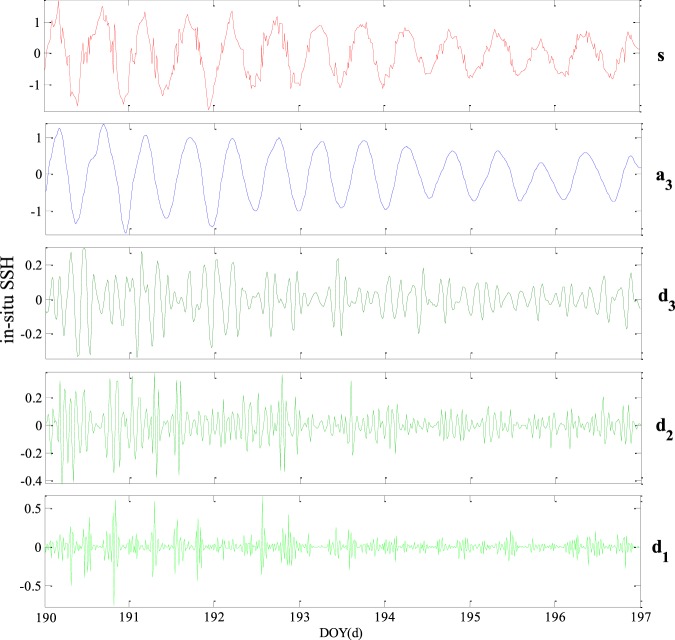
Results of wavelet decomposition of Db6 wavelet under 3^rd^ level.

Each signal has the following relationship: S = A1 + D1; A1 = A2 + D2; A2 = A3 + D3; A3 = A4 + D4. As an example of decomposition, Fig. 5 shows the results of wavelet decomposition under Db6 wavelet with decomposition level 3, and the original signal S is the *in-situ* SSH estimation of this paper. In addition, the relationship of each signal is: S = a3 + d3 + d2 + d1, a3 = d3 + d2 + d1.

**Figure 6:**
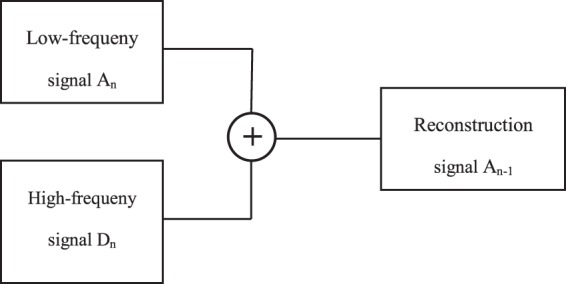
Wavelet reconstruction filter model.

The reconstruction is given by

16 $${A}_{j}[f(t)=\sum _{k}h(2t-k){A}_{j+1}[f(t)]+\sum _{k}g(2t-k){D}_{j+1}[f(t)]]$$

where *h* and *g* represents the wavelet low pass and high pass reconstruction filter, respectively. Other symbols are the same as Eq. (15). Figure 6 shows the wavelet reconstruction filter model.

One important step of wavelet de-nosing is choosing the appropriate wavelet function *W*_*ψ*_*f*(*a*, *b*). Wavelet functions such as Daubechies (dbN), Haar, and Symlets (SymN) are commonly used for wavelet analysis. To choose a suitable wavelet function, the performance of db6, Haar and Sym2 at different levels are analyzed in section “Improved SSH estimates with Wavelet De-noising”.

## Data Introduction

### GPS observations

To verify the feasibility and effectiveness of the proposed GNSS-R based SSH estimation method, the observation data of MAYG station (latitude: −12.78°, longitude: 45.26°, and height: −16.35 m), which is located in Mayotte, France near the Indian Ocean, are used. The TRIMBLE NETR9 receiver and TRM59800.00 antenna is installed in MAYG station, and the data sampling rate is 1 Hz. Because MAYG is one of the Multi-GNSS Experiment (MGEX) stations, so the data of GPS, GLONASS and BDS signals are all recorded. The navigation and observation data can be downloaded from the IGS website (http://www.igs.org/). The SNR data of BDS (L1, L5, L7; also called B1, B2, and B3), GPS (L1, L2, L5), and GLONASS (L1, L2) over a period of seven days from DOY 190 to DOY 196 in 2017 were used to evaluate the proposed method. The corresponding elevation angles range between 5°~20°, whereas the corresponding azimuth angles are between the values 20°~80° and 110°~170°, respectively. The elevation and azimuth angle selection are based on the fact that the multipath signal is strong at low elevation angle. In contrast, the reflected signals from the sea surface are of interest.

### Tide gauge observations

The observation data produced by the Dzaoudzi tide gauge, which is about ten meters from the MAYG station, were used as the ground-truth data for evaluating the performance of the proposed SSH estimation method. The sampling rate of the Dzaoudzi tide gauge data is 1 min, which can be downloaded from the Intergovernmental Oceanographic Commission (IOC) website (http://www.ioc-unesco.org/).

## Results and Discussion

### SSH estimation results with Multi-GNSS

**Figure 7:**
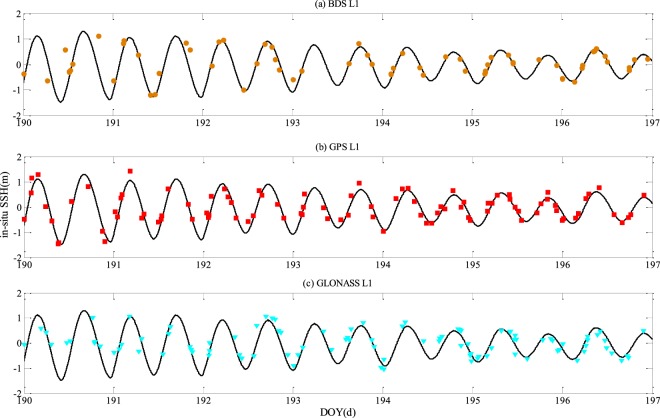
Initial *in-situ* SSH estimation results respectively produced by (**a**) BDS-R, (**b**) GPS-R, and (**c**) GLONASS-R with different frequencies The black solid line indicates the time series derived from Dzaoudzi tide gauge.

Figure 7 shows the *in-situ* SSH estimation results using the L1 signals of GPS, GLONASS, and BDS. The horizontal axis represents the DOY, and the tide gauge observations (black solid line) are also shown for comparison. The GNSS-R based SSH estimates are represented by solid circle, solid square, and solid inverted triangle, respectively for signals of three different satellites.

**Figure 8:**
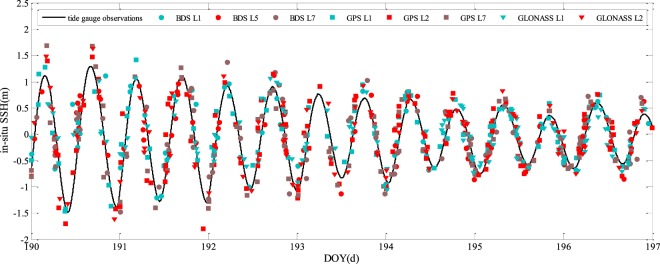
Initial *in-situ* SSH estimation results respectively produced by BDS-R, GPS-R, and GLONASS-R with different frequencies.

As can be seen from Fig. 7, there is a significant daily periodicity of *in-situ* SSH changes observed by tide gauge, which is mainly caused by tide and wind variability, as well as sea level pressure variability. There is a good agreement between tide gauge SSH observations and SSH estimates obtained by the GNSS-R methods over a period of seven days. Only a limited number of GNSS satellites can be seen over a certain period of time. Thus, SSH estimations are not uniformly distributed. In some cases, no *in-situ* SSH estimates are available over a quite long time interval. In general, the number of GPS satellites is the largest among the numbers of GPS, BDS, and GLONASS satellites. So, GPS-R has the highest temporal resolution for SSH estimation on average. Figure 8 shows the combined results of SSH estimates with all available GNSS satellites and frequencies. It is interesting to note that the time resolution of SSH is greatly improved.

**Figure 9:**
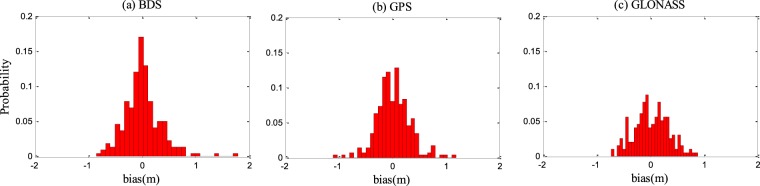
Histogram of the *in-situ* SSH estimation errors of (**a**) BDS-R, (**b**) GPS-R, and (**c**) GLONASS-R.

**Figure 10:**
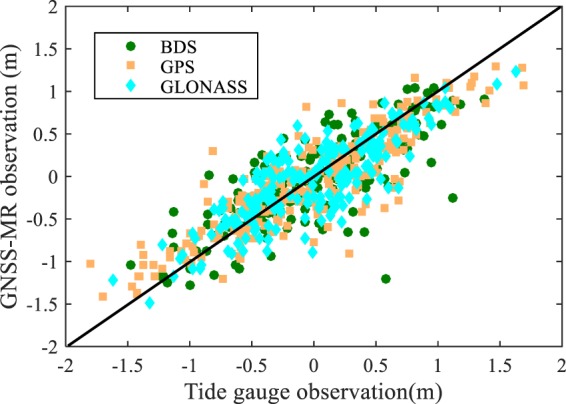
Scatter plot of sea surface height estimates by BDS-R, GPS-R, and GLONASS-R. Tide gauge data are treated as ground-truth. The black line is X = Y line.

Table 1 presents the *in-situ* SSH estimation performance in terms of RMSE and correlation coefficients with GPS, BDS, and GLONASS satellite signals of eight different frequency bands. It can be seen from Table 1 that GNSS-R with GPS L1 signal achieves the best performance, i.e., maximum correlation coefficient (0.91) and highest accuracy (RMSE = 0.23 m). Figure 9 shows the histogram of the *in-situ* SSH estimation errors of the three different navigation systems. It can be seen that the errors approximately have a normal distribution. The mean errors are all within 0.02 m, while the standard deviations are 0.31 m, 0.35 m, and 0.32 m for GPS-R, BDS-R, and GLONASS-R, respectively. The means are very close to zero, so the estimation can be considered as unbiased estimation. Figure 10 shows the scatter plot between the tide gauge observations and the GNSS-R SSH estimates. Clearly, the GNSS-R estimates are closely around the tide gauge observations. The RMSEs and correlation coefficients of the combined GNSS-R based estimates are 0.32 m and 0.83, respectively. Although the precision and correlation coefficient are slightly degraded compared with the best results using single frequency band GPS L1, the temporal resolution is increased significantly.

**Table 1:** The precisions and correlation coefficients of SSH estimation based on BDS-R, GPS-R, and GLONASS-R.

Frequency	RMSE/m	Correlation
BDS L1	0.36	0.78
BDS L5	0.32	0.80
BDS L7	0.37	0.77
GPS L1	0.23	0.91
GPS L2	0.34	0.87
GPS L5	0.34	0.88
GLONASS L1	0.31	0.80
GLONASS L2	0.33	0.84
All combined	0.32	0.83

### Improved SSH estimates with Wavelet De-noising

**Figure 11:**
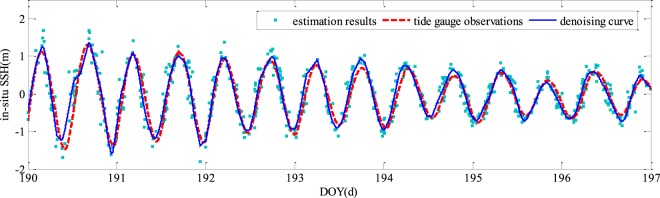
Time series of *in-situ* SSH estimates from initial results and de-noising results.

**Figure 12:**
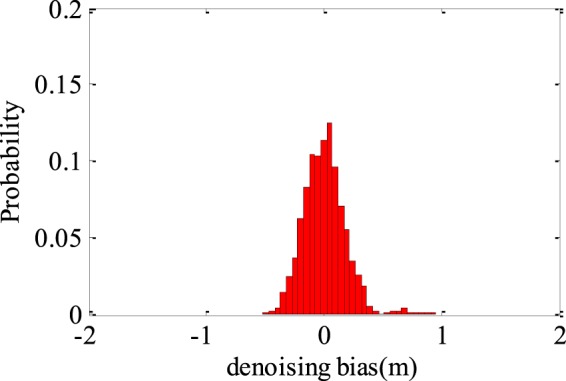
Histogram of SSH estimation errors with the de-noising method.

As observed from Fig. 8, compared with the tide gauge observations, the GNSS-R based estimates are rather noisy. In order to improve the GNSS-R based *in-situ* SSH estimation precision, we exploit the wavelet de-noising method to reconstruct the tidal waveforms. To produce the best possible wavelet de-noising results, a number of parameters should be selected appropriately^29,30^. Three of the wavelets i.e. Db6, Haar, and Sym2 are used to reconstruct the tidal waveform. The number of levels for decomposition and reconstruction is set to be 1–8. Table 2 presents the RMSEs and correlation coefficients by using the three different wavelets. It can be seen that when the decomposition level is less than 5, the three different wavelets produce very similar performance. Db6 slightly outperforms the other two wavelets and Db6 with decomposition level of 3 achieves the minimum RMSE. Meanwhile, the decomposition level is greater than 4, the performance of RMSEs and correlation coefficients are degraded, as well as Sym2 with decomposition level of 8 achieves the maximal RMSE. Therefore, only results produced by Db6 wavelet with decomposition level 3 are presented in the following. Figure 11 shows the reconstructed tidal waveforms after wavelet de-noising. For comparison, the tide gauge observations, as well as the initial GNSS-R *in-situ* SSH estimates are also presented. Basically, there is a nice match between the observed and the reconstructed tidal waveforms. The histogram of SSH estimation errors is shown in Fig. 12. By comparing with the results have shown in Fig. 9, the *in-situ* SSH estimation error is significantly reduced and their distribution is more reasonable. The correlation coefficient and RMSE of the wavelet de-noising method are respectively 0.95 and 0.19 m, which are improved by 14.5% and 40.6% over the initial multi-GNSS-R *in-situ* SSH estimation results.

**Table 2:** The precisions and correlation coefficients after wavelet de-noising with different levels.

Level	Db6		Haar		Sym2	
	RMSE (m)	Correlation coefficient	RMSE (m)	Correlation coefficient	RMSE (m)	Correlation coefficient
1	0.21	0.94	0.22	0.94	0.21	0.94
2	0.20	0.95	0.20	0.94	0.19	0.95
3	0.19	0.95	0.22	0.93	0.19	0.95
4	0.19	0.95	0.36	0.83	0.31	0.87
5	0.50	0.70	0.57	0.47	0.55	0.51
6	0.54	0.70	0.60	0.35	0.58	0.45
7	0.54	0.73	0.61	0.39	0.59	0.50
8	0.54	0.72	0.62	0.51	0.59	0.46

## Conclusions

In this paper, the SNR data of the reflected GNSS satellite signals of different frequency bands, namely BDS (L1, L5, L7), GPS (L1, L2, L5), and GLONASS (L1, L2), were employed to estimate the SSH. The initial *in-situ* SSH estimates were obtained through LSP spectral analysis on the detrended SNR time series. There is a good agreement between tide gauge SSH observations and SSH estimates obtained by the GPS, BDS and GLONASS data. Although the multi-GNSS combined precision and correlation coefficient are slightly degraded compared with GPS L1, the temporal resolution is increased significantly. Wavelet de-noising was applied to the initial *in-situ* SSH estimates to reconstruct the tidal waveform. Compared with tide gauge observations, The research findings demonstrated that the refined *in-situ* SSH estimation based on wavelet de-noising achieved a significant accuracy gain with the RMSE reduced from 0.23 m (GPS L1) and 0.32 m (multiple constellations and multiple frequencies) to 0.19 m, improving the accuracy by 17.4%, 40.6%, respectively. It is worthy of indicating that although these results are inferior to the other research (a few centimeters)^31^, the tidal change at the MAYG station in this paper is up to 3 m, while that of the previous research on other station is within 1 m. Therefore, the relative precision of our method is comparable to the previous research. Furthermore, since water levels and wave dynamics near the coast in storm conditions are very worth researching and discussing^32,33^, future study will focus on verifying the performance of this proposed method on estimating storm surge water levels.
